# Simultaneous Extraction Optimization and Analysis of Flavonoids from the Flowers of *Tabernaemontana heyneana* by High Performance Liquid Chromatography Coupled to Diode Array Detector and Electron Spray Ionization/Mass Spectrometry

**DOI:** 10.5402/2013/450948

**Published:** 2012-10-18

**Authors:** Thiyagarajan Sathishkumar, Ramakrishnan Baskar, Mohan Aravind, Suryanarayanan Tilak, Sri Deepthi, Vellalore Maruthachalam Bharathikumar

**Affiliations:** ^1^Department of Biotechnology, Kumaraguru College of Technology, Coimbatore 641049, India; ^2^Department of Biochemistry, University of Saskatchewan, Saskatoon, SK, Canada S7N 5E5

## Abstract

Flavonoids are exploited as antioxidants, antimicrobial, antithrombogenic, antiviral, and antihypercholesterolemic agents. Normally, conventional extraction techniques like soxhlet or shake flask methods provide low yield of flavonoids with structural loss, and thereby, these techniques may be considered as inefficient. In this regard, an attempt was made to optimize the flavonoid extraction using orthogonal design of experiment and subsequent structural elucidation by high-performance liquid chromatography-diode array detector-electron spray ionization/mass spectrometry (HPLC-DAD-ESI/MS) techniques. The shake flask method of flavonoid extraction was observed to provide a yield of 1.2 ± 0.13 (mg/g tissue). With the two different solvents, namely, ethanol and ethyl acetate, tried for the extraction optimization of flavonoid, ethanol (80.1 mg/g tissue) has been proved better than ethyl acetate (20.5 mg/g tissue). The optimal conditions of the extraction of flavonoid were found to be 85°C, 3 hours with a material ratio of 1 : 20, 75% ethanol, and 1 cycle of extraction. About seven different phenolics like robinin, quercetin, rutin, sinapoyl-hexoside, dicaffeic acid, and two unknown compounds were identified for the first time in the flowers of *T. heyneana*. The study has also concluded that L_16_ orthogonal design of experiment is an effective method for the extraction of flavonoid than the shake flask method.

## 1. Introduction

Herbal or medicinal plant products, in various forms, have been used to treat different illness for many hundreds of years across the world. About 70–80% of the world population, particularly in the developing countries, rely on nonconventional medicine in their primary healthcare [1]. India has a rich flora that is widely distributed throughout the country, and a large number of Indian medicinal plants are attributed with various pharmacological activities, because of diversified class of phytochemicals, but still, the efficacy of these plants are yet to be scientifically documented [2]. In general, phytochemical constituents are essential for the survival and proper functioning of plants. They provide protection against herbivores, microorganisms, and competitors, regulate growth (e.g., delaying seed germination until an appropriate time), and control pollination, fertilization, and rhizosphere environment [3]. The main secondary metabolite present in plants includes lignins, flavonoid, phenols, alkaloids, amino acid derivatives, organic acids, terpenoids, steroids, and sugar derivatives. Among different phytochemicals, flavonoid exerts a wide range of biochemical and pharmacological properties, including free radical scavenging, inhibitors of lipid peroxidation, antimicrobial, antiviral, antioxidant, antithrombogenic, hepatoprotectivity, and nephroprotectivity [4].

Plants of Apocynaceae family (Dogbane) are rich in alkaloids or glycosides, especially in seeds and latex. Some species are valuable sources of medicine, insecticides, fibers, and rubber [5]. This botanical family includes 4555 species, distributed in 415 genera [6], and the genus *Tabernaemonana *is included under this family that consists of shrubs or small trees. *Tabernaemontana heyneana* Wall. (syn.* Ervatamia heyneana*) is included in the oldest script *Amarakosam or Namalingkanusasanum* written by Amarasshimhan somewhere in between the first and sixth century AD [7]. It is known as kundalam paalai in Tamil, possesses curative properties against venereal diseases, gonorrhoea, respiratory problems, nervous disorders, diabetes, chronic bronchitis, rheumatism, cardiotonic ailments, and snake bite [8, 9]. Preliminary phytochemical screening of the ethanolic extract of the roots of *T. heyneana* Wall. revealed the presence of alkaloids, sterols, triterpenoids, resins, and flavonoids [10]. Sathishkumar et al. [11] have proved the presence of quercetin and rutin related flavonoids in the leaves of *T. heyneana*. Reports are available for the therapeutic effect of flower juice (mixed along with coconut oil) against burning sensation of eyes and improved vision [12].

Extraction is a very important process for production of flavonoid concentrate from rich sources. The traditional extraction methods possess several limitations such as time consuming, laborious, low selectivity, and yield as well as utilization of large amount of organic solvents [13]. At present, there is a renewed interest in developing new processes based on the use of different variables like temperature, solvent modifiers, and material ratio for the extraction of low molecular weight components that may be environmentally friendly and benign. Previous research documentation authenticates that temperature-assisted, enzyme-assisted, supercritical-fluid-based, and semibionic-based extractions are superior over conventional soxhlet-mediated extraction [14]. It is quite complex to predict the suitable experimental conditions for a given separation task, and therefore good experimental design becomes significantly important. Such experiments are often executed in the form of orthogonal arrays. Because of the cost efficiency, experimenters always consider as many factors as possible in a design with minimum number of runs in order to make the design saturated [15].

Although the roots and leaves have been reported to have flavonoids, no scientific information is available about the flavonoid content in flowers of *Tabernaemontana heyneana*. This paper reports about the development of extraction optimal process and high-performance liquid chromatography-diode array detector coupled with electron spray ionization/mass spectrometry (HPLC/DAD-ESI/MS) method for characterizing the chromatographic fingerprinting of flavonoid and other possible polyphenolic compounds.

## 2. Materials and Methods

### 2.1. Chemicals

 Ethanol, methanol, ethyl acetate, chloroform, heptane, acetone, AlCl_3_, ammonium hydroxide, rutin, and quercetin were obtained from SD Fine-Chem. Ltd., India. For HPLC analysis, HPLC grade acetonitrile from SD Fine-Chem. Ltd., India and formic acid from Merck, Darmstadt, Germany were obtained. For TLC analysis, silica gel G_60_ was obtained from Merck, Darmstadt, Germany.

### 2.2. Plant Material

The flowers were collected from the medicinal garden of Kumaraguru College of Technology, Coimbatore, India. The species was identified and confirmed at Botanical Survey of India (BSI), Southern Circle, Coimbatore, India (BSI/SC/5/23/06-07/Tech. 478). About 5 g of air-dried fresh flowers was dissolved in 50 mL of the solvent (methanol, ethanol, distilled water, chloroform, heptane, and acetone) and kept in an orbital shaker for overnight (shake flask method). The residue was reextracted under the same conditions. The obtained extracts were filtered with Whatman number 1 filter paper, and the filtrate was used for total flavonoid estimation.

### 2.3. Experimental Design of Extraction Process

The main factors that affect the extraction of flavonoid like temperature, extraction time, material ratio (weight of the flowers (g): volume of the solvent (mL)), solvent modifier (%), and the number of extraction cycles were investigated. The factors and the experimental design were slightly modified according to the type of the solvent used. The optimum extraction conditions were determined by L_16_ (4^5^) orthogonal design of experiment using ethanol as extracting solvent (Table 1). For ethyl acetate-mediated optimization, L_9_ (3^4^) orthogonal design of experiment was adopted (Table 2). A single-factor analysis of variance (one-way ANOVA) was adopted to investigate the effect of each factor in the extraction of flavonoid. GraphPad Prism 5 trial version software was used to carry out the statistical analysis.

### 2.4. Estimation of Total Flavonoid Content (TFC) by Aluminium Chloride Method

TFC was estimated spectrophotometrically proposed by Zhishen et al. [16] with slight modifications. To 0.1 mL of the flower extract, distilled water was added to make the volume to 5 mL. To this added 0.3 mL 5% NaNO_2_, after five minutes, added 3 mL of 10% AlCl_3_ and mixed well. Six minutes later, 2 mL of 1 M NaOH was added and the absorbance was measured at 510 nm. Rutin was used as a standard for constructing the calibration curve. 

### 2.5. Identification of Flavonoid by Thin Layer Chromatography (TLC)

The glass plates (20 × 20 cm) were coated with silica gel G_60_ (0.2–0.3 mm thick and 32 g/60 mL distilled water) and were dried at room temperature. The dried plates were activated at 100°C for 30 minutes in an oven and brought to room temperature. About 20 *μ*L of optimal extract along with standard markers (rutin and quercetin) was spotted 1.5 cm far from the edge of the plate. These glass plates were developed one dimensionally in an air tight chromatography chamber containing about 200 mL of mobile phase solvent mixture consists of ethyl acetate-ethanol-water (5 : 1 : 5, v/v/v). The developed plates were air dried and visualized under UV light at 365 nm after the application of liquid ammonia for the identification of flavonoid [17].

### 2.6. Isolation of Flavonoid by Preparative Thin Layer Chromatography (PTLC)

The glass plates (20 × 20 cm) coated with silica gel G_60_ (0.5–1.0 mm thick and 46 g/85 mL distilled water) were activated at 100°C for 30 minutes in an oven and brought to room temperature. The procedure was repeated as same as that of TLC [18].

### 2.7. FT IR Analysis

The PTLC flower eluates were mixed with 200 mg KBr (FT-IR grade) and pressed into a pellet. The sample pellet was placed into the sample holder, and FT-IR spectra were recorded in the range 4000–450 cm^−1^ in FT-IR spectroscopy (PERKIN ELMER FT-IR Spectrometer, USA).

### 2.8. HPLC-DAD-ESI/MS Analysis

The high-performance liquid chromatography-electrospray mass spectrometry (HPLC-MS) experiment was performed on THERMO Finnigan LCQ Advantage max ion trap mass spectrometer (USA) having connected Finnigan Surveyor HPLC system. The column was Waters ODS-2, 250 × 4.6 mm, and id., 5 *μ*m. The mobile phase A was made up of acetonitrile, while B was made of 0.1% formic acid (pH 4.0, adjusted with ammonium hydroxide) aqueous solution. The gradient elution was performed at 0.5 mL/min with an initial condition of 12% of mobile phase A and 88% of mobile phase B for 5 min. The mobile phase A was increased to 25%, and the elution was performed for 10 min, and then elution was performed for 10 min by a linear increase of mobile phase A to 60% and again for 5 min with an increase to 100% [19]. The eluates were monitored by PDA (multiwavelength) detector at 260 nm. About 20 *μ*L of the PTLC flower eluates was introduced into the ESI source through Finnigan surveyor autosampler. The mass spectra were scanned in the range 150–750 *m*/*z,* and the maximum ion injection time was set 200 nS. Ion spray voltage was set at 5.3 KV and capillary voltage 34 V. The MS scan ran up to 60 min, and the data reductions were performed by Xcalibur 1.4 SRI.

## 3. Results and Discussion

### 3.1. Extraction of Flavonoid from the Flowers of *T. heyneana* Using Shake Flask Method

**Figure 1:**
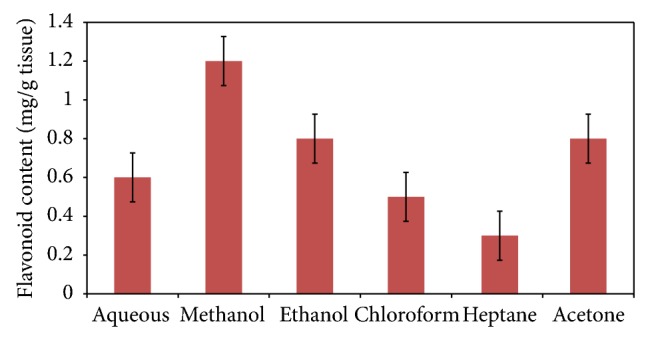
Effect of different solvents in the extraction of flavonoid from the flowers of *Tabernaemontana heyneana *Wall.

The amount of total flavonoid present in the flowers was depicted in Figure 1. Among different solvents utilized for the extraction of flavonoid, methanol was proved to be the best (1.2 ± 0.13 mg/g tissue), and heptane was observed as poor solvent (0.3 ± 0.11 mg/g tissue). Ethanol and acetone were found to be as moderate solvents in the extraction of flavonoid (0.8 ± 0.16 mg/g tissue). The standard calibration curve constructed using rutin has proved a significant positive correlation (*r* ^2^ = 0.995).

The first and foremost step in any analysis protocol is the extraction of target compound from the source material. The extraction of flavonoid can be influenced by various factors such as choice and polarity of the solvent, temperature, pressure, matrix type, solvent modification, particle size, and extraction time [20]. Previous reports have explored and revealed that various solvents like methanol, ethanol, acetone, water, ethyl acetate, ether, and, to a limited level, dimethyl sulfoxide, propanol, butanol, and their consortium have been used for the extraction of phenolic compounds [20, 21]. Our present investigation has well agreed with the previous reports explored about the extraction of flavonoid content in some fruits and vegetables by the adoption of alcoholic and organic solvents. Similarly, the results were also well corroborated with the previous investigations about the shake flask method and seemed to be inferior in the recovery of total phenolics and flavonoid content [21, 22]. 

### 3.2. Optimization of Flavonoid Extraction Using Orthogonal Design of Experiment

The optimization of flavonoid extraction in the flowers using 65–95% aqueous ethanol has yielded about 80.1 mg/g tissue (66.8 fold increase compared to an initial yield about 1.2 mg/g tissue) and ethyl acetate (20.5 mg/g tissue, 17.1 fold increase). The optimal extraction conditions of flavonoid recovery using ethanol were found as a material ratio of 1 : 20 (D_4_), temperature 85°C (A_4_), 75% ethanol concentration (C_2_), 1 time of extraction cycle (E_1_), and 3 hrs extraction duration (B_3_), that is, D_4_ > A_4_ > C_2_ > E_1_ > B_3_ (Table 3). Similarly, the optimal process of flavonoid recovery using ethyl acetate was proved to be a temperature 80°C (A_3_), material ratio of 1 : 20 (C_3_), 2 times of extraction cycle (D_2_), and 1 hr extraction duration (B_1_), that is, A_3 _> C_3 _> D_2 _> B_1_ (Table 4). The results analyzed in the form of range analysis and one-way ANOVA have revealed that material ratio (significant at 5%, (*P* < 0.05), and ethanol-mediated optimization) and temperature (significant at 1%, (*P* < 0.01), and ethyl-acetate-mediated optimization) were significant variables for the extraction and recovery of flavonoid content. Overall analysis has proved that aqueous ethanol-mediated optimal process is the best one compared to that of ethyl acetate.

Normally, the conventional solvent-mediated extraction process may depend upon conductive and convective processes to induct thermal energy into the system, and thereby, create a prolonged extraction time and increase the risk of thermal decomposition of flavonoid. This may be overcome by certain experimental approaches like temperature-assisted extraction, pressurized hot water extraction, and central composite design (response surface methodology (RSM)) which has confirmed that variables like temperature, ethanol concentration, extraction time, and material ratio were the major influencing factors in the process [23]. 

### 3.3. Effect of Temperature in the Extraction of Flavonoid

**Figure 2:**
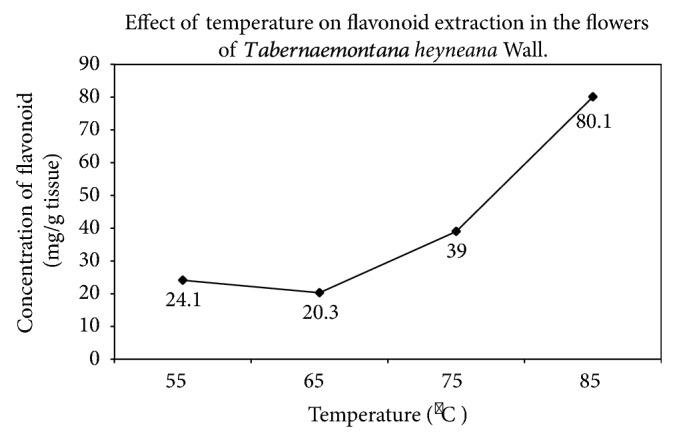
Effect of temperature on flavonoid extraction using aqueous ethanol in the flowers of *Tabernaemontana heyneana* Wall.

The contents of flavonoid gradually increased with a rise in the temperature in a range of 55°C to 85°C with a 10°C temperature interval (a slight decrease was observed between 55°C and 65°C) in ethanol-mediated optimization, and a similar pattern has been observed in ethyl-acetate-mediated optimal process (gradual and steep increase in flavonoid content from 60°C to 80°C) (Figure 2). Our present investigation was well accorded with the report documented by Sathishkumar et al. [11], on the extraction optimization of flavonoid in the leaves of *T. heyneana* Wall. According to Shi et al. [24], higher temperature would cause softening of the plant tissue, disrupting the interactions between phenolic compounds and protein or polysaccharides, and increasing the solubility of the phenolic compounds, which improves the rate of diffusion, thus giving a higher rate of extraction. On one hand, higher temperature can accelerate the solvent flow and thus increase the flavonoid content, and on the other hand, it can decrease the fluid density and viscosity, an increase in solute spread ability that could be responsible for an increase in the solvating power because of the increase in the solute vapor pressure [25]. 

### 3.4. Effect of Material Ratio in the Extraction of Flavonoid

The contents of raw flavonoid extracted from the flowers were maxima at 1 : 20 material ratio in both the solvents-mediated optimization. An entirely different contradictory result was documented by Sathishkumar et al. [11] about the optimal value of material ratio of the extraction of flavonoid from the leaves of *T. heyneana*, and this variation is probably because of the difference in the phytochemical distribution. Generally, when the solvent volume was increased, it can cause excessive swelling of the material by water and absorbing of the effective constituent [26]. A similar study performed by Xiao et al. [27] has showed that higher solvent volume may give lower yield which is totally inverse when compared with conventional extraction techniques. In contrast, an investigation made by Chen et al. [28] suggested that for a fixed amount of raw material, the more volume of solvent used, the more dilute effect in the solvent side. This gave a larger concentration difference between the interior of the plant cells and the external solvent, thus a faster extraction rate could be obtained. The basic mechanism is that the increasing ratio of solvent to raw material could decrease solution concentration difference inside and outside plant cells, which consequently prompted diffusion rate of solute particles and made more flavonoid molecules to enter the solution.

### 3.5. Effect of Ethanol in the Extraction of Flavonoid

The contents of flavonoid extracted by 75% ethanol reached maxima, and further increase in ethanol concentration may not yield increased flavonoid content. Usually, addition of a small amount of a liquid modifier can enhance significantly the extraction efficiency and, consequently, reduce the extraction time and improve the recovery of different types of natural products from plant materials [29]. Ethanol was selected as a right choice because it is environmentally benign, relatively safe to human health and interacts with the flavonoid probably through noncovalent interactions and promotes a rapid diffusion into the solution. Eventhough alone water can extract maximal flavonoid, more proteins and polysaccharides may be extracted along with it, and removal of water from the system is not cost effective, and therefore, aqueous ethanol can act as suitable extracting agent [24].

### 3.6. Effect of Extraction Time in the Extraction of Flavonoid

The optimal time duration of flavonoid extraction was found to be 3 hrs for aqueous ethanol and 1 hr for ethyl acetate, respectively. The contents of flavonoid extracted for 3 hrs reached maxima, and prolonged extraction may not yield an increased content. There is a kind of fluctuation (increase and decrease) in the flavonoid content extracted in function with time duration has been noticed in aqueous ethanol-mediated optimization. This is probably because of the synergistic effect of other parameters involved and degradation of flavonoid due to thermal-based oxidation. Increase in time also led to an increase in the adhesion of diffused particles (flavonoid) around the walls of the supporting material like glass or plastic tubes that may hinders the extraction process [30]. Similar reports made by [14, 31–33] have revealed that 2-3 hrs was the optimal extraction time and was well associated with the present investigation.

### 3.7. Effect of Number of Extraction Cycles in the Extraction of Flavonoid

The optimum number of extraction cycles for flavonoid extraction was found to be 1 cycle for aqueous ethanol and 2 cycles for ethyl acetate, respectively. The effect of repeated and successive extractions of the residue is termed as extraction cycles. During each cycle the residue was taken back and reextracted using fresh solvent under the conditions mentioned in the design [27]. The contents of raw flavonoid decreased gradually with the number of extraction cycles for aqueous ethanol-mediated optimization, and a similar pattern with slight modification has been observed for ethyl-acetate-mediated optimal process.

### 3.8. Analysis of Flavonoid by Thin Layer and Preparative Thin Layer Chromatography Techniques

TLC plates developed and sprayed with liquid ammonia revealed the presence of flavonoid glycosides (tentative rutin related compounds, bright yellow-brown color), flavonols (tentative quercetin-related compounds, bright yellow color), tentative phenolic acids (blue color), and certain other unknown phenolic compounds. The *R*_*f*_ values of rutin and quercetin were found to be 0.96 and 0.94, respectively. The *R*_*f*_ values of tentative rutin- and quercetin-related compounds were found to be 0.97 and 0.91, respectively. Thin layer chromatography (TLC) has frequently been used for the separation and the quantitative or semiquantitative analysis of natural compounds. TLC has some advantages such as rapidity, easiness, and cheapness. 

**Figure 3:**
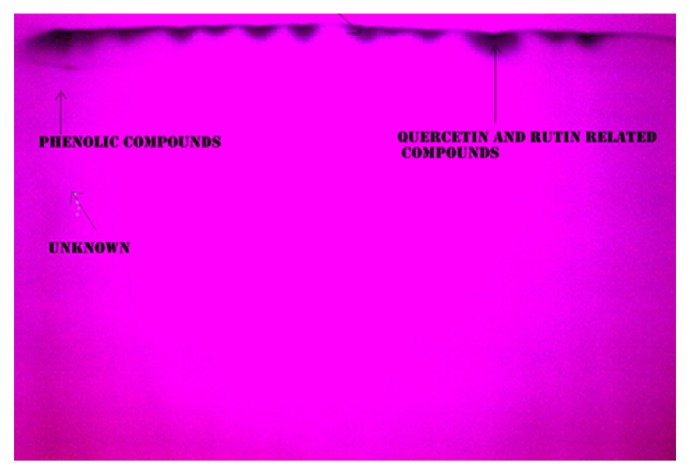
Isolation of flavonoids from optimal ethanolic extract by PTLC and visualized under far UV light.

An observation made by [16, 18, 34] regarding the identification of flavonoids and phenolic acids under far UV light has been well documented and agreed with the present results. Similarly, studies carried out by [35] have substantiated that the calculated *R*_*f*_ values of flavonoid and phenolic acids in any species may not be unique and purely depend upon the composition of the mobile phase used in the TLC. The overall results have well proved the presence of flavonols, flavonol glycosides, and phenolic acids in the flowers of *T. heyneana*. Likewise, based upon the PTLC technique, about two strong and one medium spots that have been tentatively predicted as rutin, quercetin, and phenolic acid-related compounds were successfully eluated from flowers (Figure 3). All the present investigation was well agreed by the research report documented early [11].

### 3.9. FT IR Analysis

**Figure 4:**
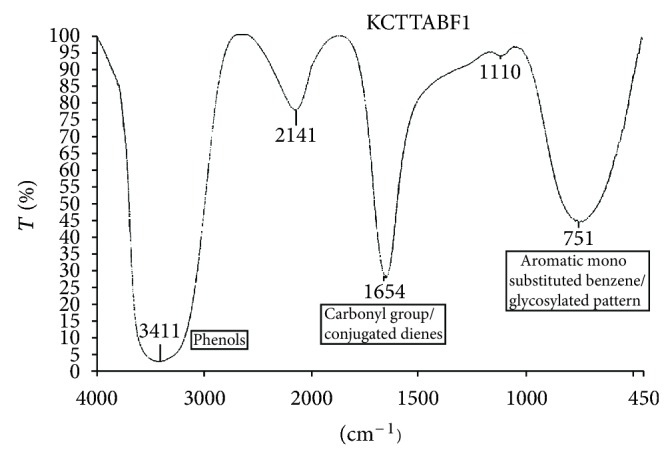
FT IR spectrum of PTLC flower eluate.

A review report by [36] has explained that a spectral range between 1185 cm^−1^ and 965 cm^−1^ showed a strong indication of C–O–C and C–OH vibrations, and absorption band between 900 cm^−1^ and 500 cm^−1^ indicates the presence of sugar moiety (glycosylation), and a spectral range at 1645 cm^−1^ proved the presence of dienes (C=C). Similarly, it has documented that a spectral band at 3400 cm^−1^ showed a broad and strong indication of presence of –OH group, exclusively phenols [37]. All these previous documentations have well correlated with the present results proving the presence of phenolic compounds that includes flavonoid and phenolic acids in flowers (Figure 4).

#### 3.9.1. HPLC-DAD-ESI/MS Analysis

The results of HPLC-DAD-ESI/MS analysis of PTLC flower eluates have revealed about 7 different compounds that include quercetin, Kaempferol-3-O-robinoside-7-O-rhamnoside (robinin), rutin, di-caffeic acid, and sinapoyl-hexoside. The investigated results with various peaks of PTLC eluates and their respective mass spectral details were depicted in Table 5.

**Figure 5:**
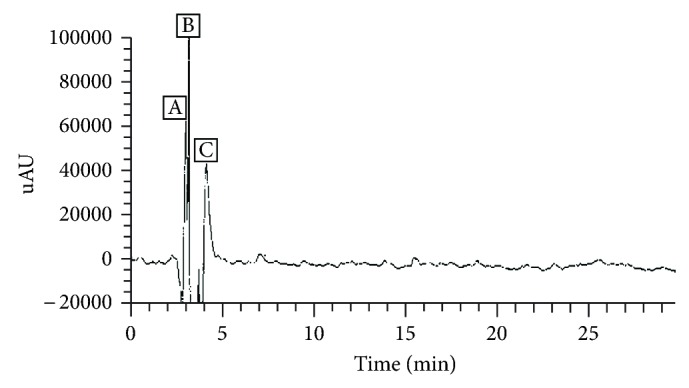
HPLC-DAD chromatogram of PTLC eluate 1. Peak 1 corresponds to unknown phenolic compound, peak 2, to dicaffeic acid, and peak 3 to kaempferol-3-O-robinoside-7-O-rhamnoside/robinin.

The liquid chromatography (LC) of PTLC flower eluate 1 has recorded three peaks A, B and C at a retention time (*R*_*t*_) of 2.55, 3.54, and 5.27, respectively (Figure 5). The Mass spectra (MS) have confirmed about three different compounds at parent ion *m/z* 340, 663, and 741 confirming the presence of dihydrocaffeic acid (RA-100%), unknown phenolic compound (RA-100%) and kaempferol-3-O-robinoside-7-O-rhamnoside (robinin) (RA-100%), respectively.

**Figure 6:**
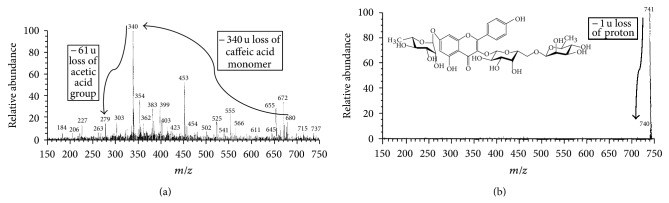
(a) Full scan ESI/MS spectra of parent ion of dicaffeic acid dimer at *m/z* 680 and fragment ions at *m/z* 340 (caffeic acid monomer), *m/z* 279, and *m/z* 184. (b) Parent ion of protonated form of kaempferol-3-O-robinoside-7-O-rhamnoside (robinin) at *m/z* 741 and [M + H]^2−^ *m/z* 740.

The MS spectra of peak B showed [M + H]^2−^ at *m/z* 341, and fragment at *m/z* 184 (protonated form of caffeic acid) proved the presence of dicaffeic acid (dimer of caffeic acid). The fragmention at *m/z* 279 denoted the loss of acetate group. The MS spectra recorded at *m/z* 680 have proved the presence of dimer adduct of caffeic acid (Figure 6(a)). All the present investigation was well correlated with the results documented early [38]. Similarly, initial research work carried out by Sakushima et al. [39] by fast atom bombardment method has revealed the fragmentation mode of several flavonoid glycosides. From their studies, robinin was found to possess the parent ion at *m/z* 741 and the fragment ion *m/z* 433/287. In our present investigation, the parent ion as [M − H]^+^ was found as *m/z* 741, and the deprotonated form [M − H]^−^ was *m/z* 740 (Figure 6(b)).

**Figure 7:**
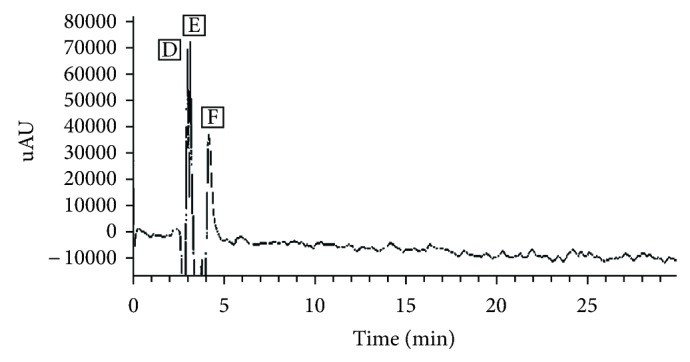
HPLC-DAD chromatogram of PTLC eluate 2. Peak 1 corresponds to sinapic acid-hexoside (hydroxycinnamic acid sugar derivative), peak 2 to unknown phenolic compound, and peak 3 to rutin.

The liquid chromatography (LC) of PTLC flower eluate 2 has recorded three peaks D, E, and F at a retention time (*R*_*t*_) of 3.19, 3.36, and 3.69, respectively (Figure 7). The mass spectra (MS) have confirmed about three different compounds at parent ion *m/z* 383, 525, and 610 confirming the presence of sinapoyl-hexoside (RA-100%), unknown phenolic compound (RA-90%), and rutin (RA-100%), respectively.

**Figure 8:**
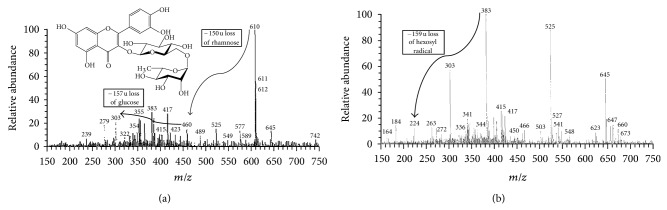
(a) Full scan ESI/MS spectra of parent ion of rutin at *m/z* 610 and fragment ions at *m/z* 460 (loss of rhamnose moiety), and *m/z* 303 (loss of glucose moiety that yield quercetin). (b) Parent ion of sinapoyl-hexoside at *m/z* 383 and [M – H − 159]^3−^ at *m/z* 224.

A study made by Dubber et al. [40] has successfully explained the fragmentation pattern of rutin obtained from *Ginkgo biloba*. In this mechanism, rutin (*m/z* 609.12) gave rise to fragment ion at *m*/*z *301 (aglycone portion of rutin, quercetin) with the corresponding loss of the rutinose unit (rhamnose plus glucose moieties) and subsequent retrocyclization of the C-ring (between bonds 1 and 2) leading to the A^−^ fragment with *m/z* 179. A most alike fragmentation of rutin has been observed in the present studies; that is, parent ion *m/z* 610 was fragmented at *m/z* 460 with the loss of −150 u ((M + H – 150), rhamnosyl moiety), and further fragmentation with the loss of −157 u ([M + H – 150 – 157], glucose moiety) yields quercetin at *m/z* 303 (Figure 8(a)). Similarly, the study carried out by Ferreres et al. [41] has explained the fragment pattern of sinapoyl-hexoside. The deprotonated parent ion *m/z* 385 has been fragmented as *m/z* 223 with the loss of −162 u (hexosyl radical) and at *m/z* 205 with the loss of −180 u (hexosyl radical + water). The current results have revealed the presence of sinapoyl-hexoside at parent ion *m/z* 383 that was fragmented as *m/z* 224 with the loss of −159 u (hexosyl radical) were in well concordance with the above said report (Figure 8(b)).

**Figure 9:**
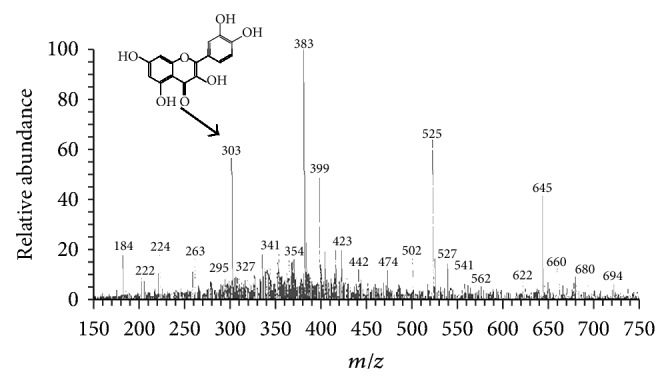
Full scan ESI/MS spectra of parent ion of quercetin at *m/z* 303.

The liquid chromatography (LC) of PTLC flower eluate 3 has recorded a single peak G at retention time (*R*_*t*_) of 2.50. The mass spectra (MS) have recorded the presence of quercetin (RA-55%) at parent ion *m/z* 303 (Figure 9). Several research reports on the LC-MS analysis of quercetin have documented as *m/z* 303. The overall results have proved that the flowers of *T. heyneana* possess appreciable quantity of flavonoids and phenolic acids.

## 4. Conclusion

In summary, the flavonoid extraction process was successfully optimized by aqueous ethanol-mediated L_16_ orthogonal design of experiment, and the components were characterized by HPLC-DAD-ESI/MS analysis. The optimal conditions were found to be 85°C, 3 hrs extraction duration, 75% ethanol concentration, a material ratio 1 : 20, and 1 time of extraction cycle. The HPLC-DAD-ESI/MS analysis performed in the PTLC flower eluates has identified compounds like rutin, quercetin, robinin, sinapoyl-hexoside, and two unknown phenolics.

## Figures and Tables

**Table 1:** Different variables for optimal extraction of flavonoids from the flowers of *T. heyneana* (aqueous ethanol as extraction solvent, 4^5^-L_16_ design).

Levels	A	B	C	D	E
	Temp. (°C)	Ext. tim. (hrs)	Ethanol (%)	Material ratio (g : mL)	No. of ext. cycles
1	55	1	65	1 : 05	1
2	65	2	75	1 : 10	2
3	75	3	85	1 : 15	3
4	85	4	95	1 : 20	4

**Table 2:** Different variables for optimal extraction of flavonoids from the flowers of *T. heyneana* (ethyl acetate as extraction solvent, 3^4^-L_9_ design).

Levels	A	B	C	D
	Temp. (°C)	Ext. tim. (hrs)	Material ratio (g : mL)	No. of ext. cycles
1	60	1	1 : 10	1
2	70	2	1 : 15	2
3	80	3	1 : 20	3

**Table 3:** Flavonoid yield in the flowers of *T. heyneana* using aqueous ethanol as extracting solvent.

Exp.	A	B	C	D	E	Flavonoid yield (mg/g)
1	1	1	2	3	4	21.8
2	1	2	1	4	3	24.1
3	1	3	4	1	2	10.4
4	1	4	3	2	1	19.2
5	2	1	1	1	1	10.7
6	2	2	2	2	2	17.1
7	2	3	3	3	3	18.9
8	2	4	4	4	4	20.3
9	3	1	3	4	2	39.0
10	3	2	4	3	1	16.2
11	3	3	1	2	4	7.0
12	3	4	2	1	3	14.6
13	4	1	4	2	3	26.3
14	4	2	3	1	4	10.5
15	**4**	**3**	**2**	**4**	**1**	**80.1**
16	4	4	1	3	2	27.2
*K*_1_	18.9	24.4	17.3	11.6	31.6	
*K*_2_	16.8	17.0	33.4	17.4	23.4	
*K*_3_	19.2	29.1	21.9	21.0	21.0	
*K*_4_	36.0	20.3	18.3	40.9	15.4	
*k*_1_	4.7	6.1	4.3	2.9	7.9	
*k*_2_	4.2	4.3	8.4	4.4	5.9	
*k*_3_	4.8	7.3	5.5	5.3	5.3	
*k*_4_	9	5.1	4.6	10.2	3.9	
*R*	**4.8**	**3.0**	**4.1**	**7.3**	**4.0**	

**Table 4:** Flavonoid yield in the flowers of *T. heyneana* using ethyl acetate as extracting solvent.

Exp.	A	B	C	D	Flavonoid yield (mg/g)
1	1	1	1	1	2.8
2	1	2	2	2	3.7
3	1	3	3	3	9.4
4	2	1	2	3	6.4
5	2	2	3	1	10.4
6	2	3	1	2	8.9
7	**3**	**1**	**3**	**2**	**20.5**
8	3	2	1	3	18.2
9	3	3	2	1	11.0
*K*_1_	5.3	9.9	10	8.0	
*K*_2_	8.5	10.8	7.0	11.0	
*K*_3_	16.6	9.8	13.4	11.3	
*k*_1_	1.8	3.3	3.3	2.7	
*k*_2_	2.8	3.6	2.3	3.7	
*k*_3_	5.5	3.3	4.5	3.8	
*R*	3.7	0.3	2.2	1.1	

**Table 5:** Retention times (*R*_*t*_), molecular ions [M + H]^+^, fragment ions, and relative abundance (%) of *T. heyneana* PTLC flower eluates (flavonols, flavonoid glycosides, and phenolic acids and derivatives scanned at 260 nm (PDA)).

S. no.	Retention time (*R*_*t*_, min)	MS *m/z* [M + H]^+^	*m/z* fragment ions	Identified molecular weight	Identified compound	Relative abundance (%)
PTLC flower eluate 1						
1	2.55	663	388/372/357	663	Unknown	100
2	3.54	340	184	340	Dicaffeic acid (diCA)	100
3	5.27	741	—	741	Kaempferol-3-O-robinoside-7-O-rhamnoside/robinin	100
PTLC flower eluate 2						
4	3.19	383	224	383	Sinapic acid-hexoside (hydroxycinnamic acid sugar derivative)	100
5	3.36	525	—	525	Unknown	90
6	3.69	610	303	610	Rutin	100
PTLC flower eluate 3						
7	3.17	303	—	303	Quercetin	55
